# New atypical epidemiological profile of parvovirus B19 revealed by molecular screening of blood donations, France, winter 2023/24

**DOI:** 10.2807/1560-7917.ES.2024.29.21.2400253

**Published:** 2024-05-23

**Authors:** Marlène Guillet, Ariane Bas, Marjorie Lacoste, Céline Ricard, Catherine Visse, Valérie Barlet, Lucile Malard, Sophie Le Cam, Pascal Morel, Xavier de Lamballerie, Syria Laperche, Pierre Gallian

**Affiliations:** 1 Etablissement Français du Sang Centre Pays de Loire, Nantes, France; 2 Etablissement Français du Sang Auvergne Rhône Alpes, Lyon Décines, France; 3 Etablissement Français du Sang Occitanie, Toulouse, France; 4 Etablissement Français du Sang Hauts de France-Normandie, Loos, France; 5 LFB – Biomédicaments, Control Laboratories, Courtaboeuf Cedex, France; 6 Etablissement Français du Sang, La Plaine Saint-Denis, France.; 7 Unité des Virus Émergents (UVE: Aix-Marseille Univ-Corsica Univ-IRD 190-Inserm 1207-IRBA), Marseille, France

**Keywords:** Parvovirus B19, Blood donors, COVID-19, non-pharmaceutical interventions, plasma-derived medicinal products

## Abstract

In France, blood donations are tested in pools of 96 samples for parvovirus B19 (B19V) DNA to discard plasma for fractionation when it contains high viral loads. Between January 2015 and March 2024, B19V-positive donations decreased during the COVID-19 pandemic, followed by a strong rebound in 2023 and unusually high circulation during winter 2023/24 (ca 10 times higher December 2023–March 2024 vs the pre-pandemic period). Variations over time are probably related to measures implemented to limit SARS-CoV-2 spread.

Parvovirus B19 (B19V) is a small, non-enveloped DNA virus belonging to the *Parvoviridae* family (genus *Erythrovirus*), mainly transmitted via the respiratory route. It is endemic worldwide. In Europe, infections mostly occur in spring and early summer. Outbreaks of varying intensity spaced out over several years are observed [1]. Many European countries have since the end of 2023 reported an increase in B19V infections in children, pregnant women and blood donors [2]. 

The aim of this analysis was to describe changes in the prevalence of B19V in French blood donations between 2015 and 2024 and highlight the impact of the recent increase on blood donations. 

## Molecular screening of parvovirus B19 in French blood donations

Parvovirus B19 can be transmitted by transfusion. The duration of viraemia in blood donors has been estimated at 17.5 days (95% confidence interval (CI): 11–53) during acute infection [1]. However, prolonged DNA shedding for up to 4 years has been reported, if at low levels [3,4]. High levels of B19V DNA (up to 10^12^ IU/mL) usually observed during primary infection have been detected in plasma donations [5,6], and cases of transmission involving various types of blood products and plasma-derived medicinal products (PDMPs) have been reported [7-10]. 

To limit the risk of transmission by PDMPs, the LFB Biomédicaments (Laboratoire Français du Fractionnement et des Biotechnologies, Courtaboeuf, France), introduced nucleic acid testing (NAT) of B19V DNA (subcontracted to the French transfusion public service since 2015) to control the viral load of plasma batches before the viral inactivation/elimination steps during PDMPs production processes. In 2001, the European Pharmacopoeia made the screening of B19V by NAT mandatory in plasma pools used for the manufacture of anti-D immunoglobulin, with a maximum of 10.0 IU/µL [11]. 

In France, B19V-NAT is performed on pools of 96 donations (P96) with a threshold of 10^4^ IU/mL B19V DNA, corresponding to 10^6^ IU/mL at the single donation level. A duplex assay Procleix HAV/B19 assay (Tigris/Grifols) was used from 2015 to March 2023 and replaced by DPX Parvo/HAV (Cobas 8800/Roche) from April 2023. The positive P96 were broken down into two pools of 48 donations (P48) from 2015 to March 2023 and 16 pools of six donations (P6) from April 2023. All donations included in positive P48, or after April 2023 P6, pools were excluded from the fractionation process. Positive pools were not resolved into individual positive high-titre donations, resulting in missing data on the sex and age groups of B19V-infected blood donors. Due to pool screening and the use of a quantitative threshold, the precise prevalence of B19V DNA in the French blood donor population cannot be assessed using this strategy.

## Monitoring the prevalence of parvovirus B19 DNA in blood donations from 2015 to 2024

**Figure 1:**
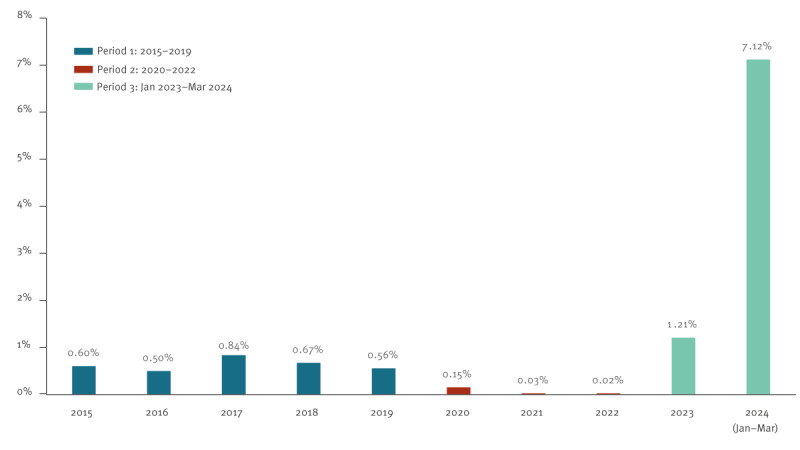
Annual prevalence of parvovirus B19 DNA-positive pools of 96 blood donations, France, January 2015–March 2024 (n = 270,508 pools tested)

Between 2015 and early 2024, 1,863 (0.69%) of 270,508 pools (corresponding to 25,968,768 donations) tested positive for B19V DNA. We identified three periods: the pre-COVID-19 period (P1: 2015–2019) in which the prevalence of B19V DNA-positive pools was 0.63% (95% CI: 0.59–0.68), the COVID-19 period (P2: 2020–2022) with a prevalence of 0.07% (95% CI: 0.05–0.09) and the post-COVID-19 period (P3: January 2023–March 2024) with a prevalence of 2.40% (95% CI: 2.24–2.56) (Figure 1). Prevalence values were significantly higher when comparing the periods P1 vs P2 (chi-squared test: p < 0.0001), P3 vs P2 (p < 0.0001) and P3 vs P1 (p < 0.0001). 

**Figure 2:**
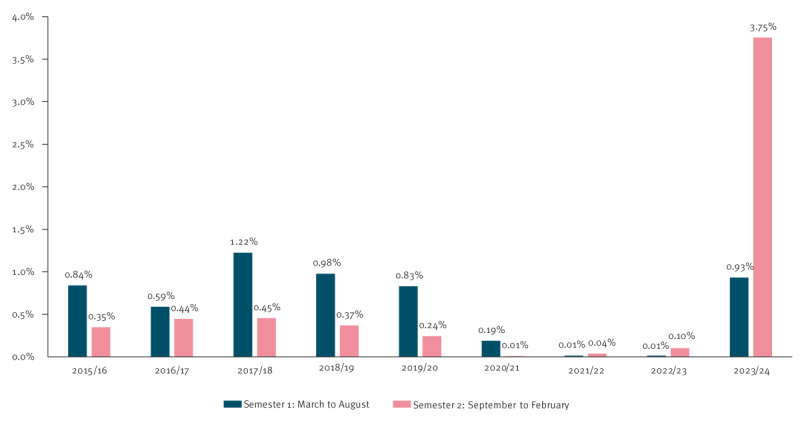
Seasonality of the prevalence of parvovirus B19 DNA-positive pools of 96 blood donations, France, March 2015–February 2024 (n = 265,016 pools tested)

We investigated the seasonality (Figure 2) by comparing semesters S1 (March to August year n) and S2 (September year n to February year n + 1). The S1 rate was significantly higher than the S2 rate during the P1 period: 0.89% (95% CI: 0.82–0.96) vs 0.37% (95% CI: 0.33–0.41) (p < 0.0001); both the S1 and the S2 rate were low during the P2 period; during the P3 period, the S1 rate was lower than the S2 rate, indicating an atypical viral circulation profile in that winter: 3.75% (95% CI: 3.44–4.07) vs 0.93% (95% CI: 0.77–1.09) (p < 0.0001).

## Parvovirus B19 prevalence in French blood donations before and during the COVID-19 pandemic

During the COVID-19 outbreak, a wide range of non-pharmaceutical interventions were implemented from March 2020 to limit the spread of severe acute respiratory syndrome coronavirus 2 (SARS-CoV-2) in the general population, such as strict lockdown (mid-March to mid-May 2020), hand hygiene measures, social distancing and mask wearing. These measures were effective in reducing the transmission of respiratory viruses such as influenza and respiratory syncytial virus [12]. When compared with the previous years, the incidence of acute respiratory infection fell by 49% during the first lockdown in France [13]. During the COVID-19 pandemic, there were no influenza epidemics in Europe [14,15] and no bronchiolitis epidemics in France [16]. The decrease in the number of B19V DNA-positive blood donation pools observed in France between March 2020 and November 2022 coincides with the implementation of COVID-19 control measures. 

A similar decrease was reported in other European countries where comparable measures had been implemented. In the Netherlands, no plasma donations with a high viral load (≥ 10^6^ IU/mL) of B19V DNA were detected between May 2020 and June 2021 [17]. In Catalonia (Spain), the regional blood bank observed a substantial decrease when comparing annual B19 positivity rates between the period from 2014 to 2019 and the years 2020 and 2021, with only three B19V DNA-positive cases detected between January 2020 and July 2021 [18]. Similarly, B19V NAT positivity rates in Canadian plasma samples decreased significantly between the pre-pandemic period (0.01%) and the pandemic period (0.0005%) [19].

From summer 2021 onwards, the decline in the COVID-19 epidemic in France led to relax distancing measures. Face masks were recommended in public places until 14 June 2021. Most of the measures including hygiene rules (hand washing and use of hydro-alcoholic gel) were applied less stringently, until they became unusual in the general population from 2022 onwards. As a result, the number of B19V DNA-positive pools increased in 2023, with rates between March and August (S1) similar to the pre-COVID-19 period.

## Atypical parvovirus B19 epidemic rebound during the winter of 2023/24

**Figure 3:**
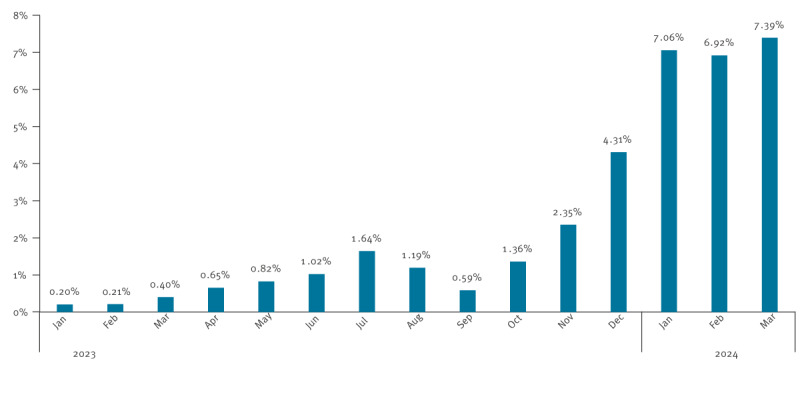
Monthly prevalence of parvovirus B19 DNA-positive pools of 96 blood donations, France, January 2023–March 2024 (n = 35,390 pools tested)

Unexpectedly, the epidemic developed during the following winter season, with B19V DNA-positivity rates almost doubling each month between September 2023 and January 2024 (Figure 3). Consequently, the rate detected between September 2023 and February 2024 was significantly higher than those of the previous half year (S1–2023) and those of the S1 half years of the pre-COVID-19 period (Figure 2). 

A similar epidemiological profile was observed in Israel where a large outbreak was identified in the general population following a low level of viral circulation during the COVID-19 years 2020 to 2022 [20]. We speculate that this atypical epidemiological pattern could be partly explained by a reduction in herd immunity in relation to the low levels of B19V circulation over the previous 2 years. To support this hypothesis, the increase in B19V DNA positivity rate among French blood donors coincided with a resumption of the B19V epidemic in the general population. Firstly, a French study showed an important increase in the number of children infected with B19V since April 2023, after the incidence had been low for 3 years [21]. In April 2024, several European countries including France reported an increase in the number of cases affecting all age groups, with children being the most affected [2]. In addition, in France, the number of serious complications of congenital infection, including miscarriage and, during the first trimester of 2024, the death of four newborns due to congenital infection, was higher than in previous years [2].

## Discussion

Parvovirus B19 infections are generally asymptomatic. When present, symptoms are usually benign and include mild rash (erythema infectiosum or fifth disease) in children and arthropathies and inflammation of other tissues in immunocompetent adults. Due to the viral tropism for erythroid progenitor cells, acute or chronic anaemia in immunocompromised patients and transient aplastic crisis in patients with sickle cell disease or chronic haemolytic anaemia may be observed. During pregnancy, foetal infection in non-immune women may cause severe complication as foetal anaemia, hydrops and pregnancy loss. 

In agreement with recommendations from the European Centre for Disease Prevention and Control, our findings suggest that the medical community should be made aware about the current unusually high levels of B19V circulation in Europe and the atypical seasonal pattern associated with this circulation which merits particular vigilance. The French public health agency recommends paying particular attention to the risk of B19V infection in immunocompromised/sickle-cell-affected children and pregnant women and avoiding all contact between vulnerable people and those infected or suspected of being infected [22]. The high-level circulation of B19V also has an impact on the number of donations excluded from plasma fractionation. Although this impact was limited by breaking down the P96-positive pools into pools of decreasing size (P48, then P6), measures such as unit pool resolution could be considered to limit plasma loss during intense and prolonged epidemics.

## Conclusion

Our data show a marked decrease in B19V detections in the French blood donor population during the COVID-19 epidemic, followed by a strong epidemic rebound in 2023 with atypical persistence during winter and high circulation levels continuing in early 2024. These variations over time are probably related to the measures implemented to limit the spread of the COVID-19 pandemic. On the basis of these observations, we suggest that it would be interesting to study in greater detail the value of monitoring the prevalence of B19V in blood donors in order to estimate the adherence to and effectiveness of measures to prevent the transmission of acute respiratory infections.

## References

[1] Zaaijer HL Koppelman MH Farrington CP. Parvovirus B19 viraemia in Dutch blood donors. Epidemiol Infect. 2004;132(6):1161-6. 10.1017/S0950268804002730 15635975 PMC2870209

[2] European Centre for Disease Prevention and Control (ECDC). Communicable disease threats report, 14-20 April 2024, week 16. Stockholm: ECDC; 2024. Available from: https://www.ecdc.europa.eu/en/publications-data/communicable-disease-threats-report-14-20-april-2024-week-16

[3] Candotti D Etiz N Parsyan A Allain JP. Identification and characterization of persistent human erythrovirus infection in blood donor samples. J Virol. 2004;78(22):12169-78. 10.1128/JVI.78.22.12169-12178.2004 15507603 PMC525065

[4] Matsukura H Shibata S Tani Y Shibata H Furuta RA. Persistent infection by human parvovirus B19 in qualified blood donors. Transfusion. 2008;48(5):1036-7. 10.1111/j.1537-2995.2008.01704.x 18454740

[5] Sun P Jiang P Liu Q Zhang R Wang Z Cao H Parvovirus B19 DNA and antibodies in Chinese plasma donors, plasma pools and plasma derivatives. PeerJ. 2023;11:e15698. 10.7717/peerj.15698 37554334 PMC10405795

[6] Williams S Ratcliff J Nguyen D Simmonds P Harvala H International Survey Group. Detection frequencies and viral load distribution of parvovirus B19 DNA in blood and plasma donations in England. Transfus Med. 2022;32(5):402-9. 10.1111/tme.12893 35751630 PMC9796365

[7] Gowland P Fontana S Stolz M Andina N Niederhauser C. Parvovirus B19 passive transmission by transfusion of intercept® blood system-treated platelet concentrate. Transfus Med Hemother. 2016;43(3):198-202. 10.1159/000445195 27403092 PMC4924464

[8] Azzi A Morfini M Mannucci PM. The transfusion-associated transmission of parvovirus B19. Transfus Med Rev. 1999;13(3):194-204. 10.1016/S0887-7963(99)80033-9 10425692

[9] Marano G Vaglio S Pupella S Facco G Calizzani G Candura F Human parvovirus B19 and blood product safety: a tale of twenty years of improvements. Blood Transfus. 2015;13(2):184-96. 25849894 10.2450/2014.0174.14 PMC4385066

[10] Satake M Hoshi Y Taira R Momose SY Hino S Tadokoro K. Symptomatic parvovirus B19 infection caused by blood component transfusion. Transfusion. 2011;51(9):1887-95. 10.1111/j.1537-2995.2010.03047.x 21332725

[11] Human anti-D immunoglobulin for Intravenous Administration. In: European Pharmacopoeia. 10^th^ ed. Strasbourg: The European Pharmacopoeia Commission; 2020.Available from: https://www.edqm.eu/en/-/shutdown-of-european-pharmacopoeia-10th-edition

[12] Nawrocki J Olin K Holdrege MC Hartsell J Meyers L Cox C The effects of social distancing policies on non-SARS-CoV-2 respiratory pathogens. Open Forum Infect Dis. 2021;8(7):ofab133. 10.1093/ofid/ofab133 34322558 PMC7989184

[13] Launay T Souty C Vilcu AM Turbelin C Blanchon T Guerrisi C Common communicable diseases in the general population in France during the COVID-19 pandemic. PLoS One. 2021;16(10):e0258391. 10.1371/journal.pone.0258391 34634090 PMC8504745

[14] Sanz-Muñoz I Tamames-Gómez S Castrodeza-Sanz J Eiros-Bouza JM de Lejarazu-Leonardo RO. Social distancing, lockdown and the wide use of mask; a magic solution or a double-edged sword for respiratory viruses epidemiology? Vaccines (Basel). 2021;9(6):595. 10.3390/vaccines9060595 34205119 PMC8228489

[15] Bernard-Stoecklin S, Équipes de surveillance de la grippe. Surveillance de la grippe en France, saison 2022-2023. [Influenza surveillance in France, 2022-2023 season. Bull Épidémiol Hebd. 2023;(19):382-97. French. Available from: http://beh.santepubliquefrance.fr/beh/2023/19/2023_19_1.html

[16] Santé Publique France. Bronchiolite. Bilan de la surveillance 2022-2023. [Bronchiolitis. Surveillance report 2022-2023]. Saint-Maurice: Santé Publique France; 2023. French. Available from: https://www.santepubliquefrance.fr/maladies-et-traumatismes/maladies-et-infections-respiratoires/bronchiolite/documents/bulletin-national/bulletin-epidemiologique-bronchiolite.-bilan-de-la-surveillance-2022-2023

[17] Molenaar-de Backer MW Hogema BM Koppelman MH van de Laar TJ Slot E Zaaijer HL. Lower incidence of parvovirus-B19 infections in Dutch blood donors during SARS-CoV-2 Pandemic. Microbiol Spectr. 2021;9(2):e0025321. 10.1128/Spectrum.00253-21 34523999 PMC8565513

[18] Sauleda S Piron M Bes M Martinez-Llonch N Puig L. Changes in parvovirus B19 positivity rates in plasma units for fractionation: An unexpected effect of non-pharmaceutical interventions against COVID-19? Vox Sang. 2022;117(4):626-7. 10.1111/vox.13229 34877666

[19] Drews SJ Charlton C O’Brien SF Burugu S Denomme GA. Decreasing parvovirus B19 and hepatitis A nucleic acid test positivity rates in Canadian plasma donors following the initiation of COVID-19 restriction in March 2020. Vox Sang. 2024;vox.13616. 10.1111/vox.13616 38482941

[20] Patalon T Saciuk Y Trotzky D Pachys G Ben-Tov A Segal Y An outbreak of parvovirus B19 in Israel. Viruses. 2023;15(11):2261. 10.3390/v15112261 38005937 PMC10674631

[21] Fourgeaud J Allali S Toubiana J Pinhas Y Frange P Leruez-Ville M Post-COVID-19 pandemic outbreak of severe parvovirus B19 primary infections in Paris, France: 10-year interrupted time-series analysis (2012-2023). J Clin Virol. 2023;167:105576. 10.1016/j.jcv.2023.105576 37633184

[22] Semaille C (directrice de publication). Epidémie d’infections à Parvovirus B-19. [Epidemic of parvovirus B-19 infections]. Saint-Maurice: Santé publique France; 2024. French. Available from: https://www.santepubliquefrance.fr/content/download/620047/4261122?version=2#:~:text=Une%20%C3%A9pid%C3%A9mie%20d'infections%20%C3%A0,atteint%20au%20mois%20de%20mars

